# Aerosol classification by dielectrophoresis: a theoretical study on spherical particles

**DOI:** 10.1038/s41598-020-67628-9

**Published:** 2020-06-30

**Authors:** Malte Lorenz, Alfred P. Weber, Michael Baune, Jorg Thöming, Georg R. Pesch

**Affiliations:** 1 Faculty of Production Engineering, Chemical Process Engineering (CVT), University of Bremen, Bremen, Germany; 2 Institute of Particle Technology, Clausthal University of Technology, Clausthal-Zellerfeld, Germany; 3 MAPEX Center for Materials and Processes, University of Bremen, Bremen, Germany

**Keywords:** Chemical engineering, Characterization and analytical techniques

## Abstract

The possibilities and limitations using dielectrophoresis (DEP) for the dry classification of spherical aerosol particles was evaluated at low concentrations in a theoretical study. For an instrument with the geometry of concentric cylinders (similar to cylindrical DMA), the dependencies of target particle diameter $d_{{\text {P}}}^*$, resolution, and yield of the DEP classification on residence time, applied electric field strength, and pressure of the carrier gas were investigated. Further, the diffusion influence on the classification was considered. It was found that $d_{{\text {P}}}^*$ scales with the mean gas flow velocity $u_{{\text {gas}}}$, classifier length *L*, and electric field strength *E* as $d_{{\text {P}}}^*\propto (u_{{\text {gas}}}/L)^{0.5}E^{-1}$. The resolution of the classification depends on the particle diameter and scales proportionally to ${d_{{\text {P}}}^*}^{1.3}$. It is constrained by the flow ratio $\beta$ (i.e., sheath gas to aerosol flow), electrode diameters, and applied electric field strength. The classification yield increases with the ratio of the width of the extended outlet slit $s_{{\text {e}}}$ to the diffusion induced broadening $\sigma _z$. As expected, resolution and yield exhibit opposite dependencies on $s_{{\text {e}}}/\sigma _z$. Our simulations show that DEP classification can principally cover a highly interesting particle size range from 100 nm to ${10}\,\upmu \hbox {m}$ while being directly particle size-selective and particle charge independent.

## Introduction

The dry classification of particles with diameters below ${10}\,\upmu \hbox {m}$ is key to obtain high-value powders with narrow size distributions. These fine particles find application in a variety of high-value products such as coatings^1^, printing products^2^, porous functional structures ^3^, particle-reinforced polymers^4^ and electrodes in electro-chemical energy storage^5^. Many established classification technologies suffer from insufficient selectivity or throughput in this size range. For instance sieving, which is a common tool in industrial separation, comes only close to this size range when performed as wet technique ^6^. When starting from a dry powder, wet sieving could lead to agglomeration and material property changes and would additionally require an uneconomical drying step following the classification. Deflection wheel classifiers exhibit a significant loss in sharpness of cut and throughput in this particle size range ^7^. Electrophoretic mobility classification relies on the defined charging of the particles, and thus particle-size-dependent charge probability distributions. Hence, particle size is not directly measured but merely the electrophoretic mobility distribution of the particles. A standard inversion procedure exists to back-calculate size distribution from electrophoretic mobility measurements. Nevertheless, exact particle size classification is not possible with this technique because particles of different size may have the same electrophoretic mobility due to their charge. A comprehensive survey concerning charging of airborne particles is given for example by Friedlander^8^, chapter 2.

In this manuscript we outline the behaviour of a particle-charge-independent and directly size-dependent classification method which attracted very little attention so far, i.e., the aerosol classification by dielectrophoresis (DEP). Dielectrophoresis is the movement of charged or *uncharged* particles in *inhomogeneous* electric fields. It is caused by the interaction of an induced dipole (or quadrupole, octupole, ...) with an electric field gradient^9^. DEP has mostly been used in liquid media^10^, for example for the manipulation, separation, and characterization of macromolecules^11^ or bio particles^12,13^. In industrial scale processes it is investigated as a measure against colloidal membrane fouling^14^ or as a separating force in particle sorting^15^ and filtration processes^16–19^.

Contrary to electrophoresis, which requires particle charging and is thus dependent on a particle charge distribution, DEP uses the inherent dielectric properties of the particles determined by the particle volume and the particle polarizability. However, depending on the goal of the separation, the influence of the material (i.e., dielectric constant and conductivity), reflected by the Clausius–Mossotti function, can be influenced thus rendering the separation material independent, for instance by controlling the humidity of the carrier gas. For example, Wang *et al.*^20^ showed that a relative carrier gas humidity of 15% changes the conductivity of glass fibers making them sufficiently polarizable for classification. In low-frequency electric fields ($<\, {200}\,\hbox {kHz}$) nearly every particle that is slightly conductive is much more polarizable than air so that the particle migration velocity does only depend on particle volume and projection area, similar to the classification in centrifugal fields. However, DEP does not require moving parts such as rotating cylinders (aerosol particle mass analyzer). DEP could therefore be used to separate particles independent of their material according to their volume and projection area or to separate particles according to their polarizability (as expressed by the Clausius–Mossotti function, e.g., separate conductive and non-conductive particles). The present study simulatively investigates the material-independent classification of spherical particles by diameter under the assumption that they are perfectly polarizable (e.g., by tuning the humidity of the carrier gas).

In a few available studies, DEP was used to separate airborne fibers according to their length ^20–24^. The so-called Baron separator is the only reported application of DEP for the separation or classification of airborne particles. It consists of two conductive cylinders, a solid one of small diameter and a hollow one of larger diameter placed concentrically around the first so that an annular gap is formed. An alternating field is applied across this gap. The classifier uses the fact that fibers (mostly independent of their cross-sectional diameter) have different dielectrophoretic mobilities according to their length. It was investigated for the classification of airborne asbestos fibers for further assessment of their health impact.

The available literature concerning the Baron separator is limited to the four journal articles and the dissertation listed above which serve as a starting point of our own investigation. In this first step we employ a less complex model of an apparatus, which follows a similar concept as the Baron separator. Inlet and outlet configurations, however, show distinct differences. We use computer-assisted calculation (particle tracking algorithms) to investigate the theoretical possibility to separate and classify airborne spherical micron, sub-micron and nano particles using dielectrophoresis in a concentric separator. An alternating electric field is applied for the process to be independent of particle charge because, within certain limits, there is no net electrophoretic particle migration at sufficiently high frequencies.

In this study, we show the possibilities and limitations (applicable particle size range, resolution, and achievable yield) of DEP classification of airborne particles which could also be used for the detection of airborne viruses. To the best of our knowledge, this is the first study addressing the classification of spherical particles in air using DEP. Previous studies only addressed length classification of fibers and were thus limited in their applicability. We investigate the influence of classifier length, volume flow, and electric field strength on the target particle diameter and assess the influence of particle diffusion and process parameters on the transfer function (resolution and yield). We suggest a very simple approach for calculating particle diffusion that does not require calculation of a vast number of random walk trajectories. This method is based on particles’ mean square displacement. A second more common method to calculate diffusion is the Langevin approach that uses a stochastic description of a random walk. We use this to validate the mean-square-displacement method.

## Model and theory

### DEP particle classifier concept

**Figure 1:**
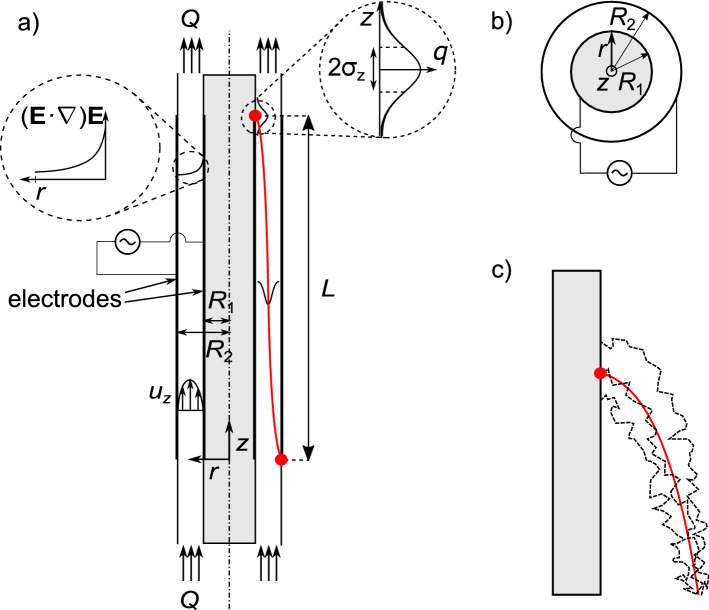
(**a**) Side view (sketch) of a dielectrophoretic aerosol classifier. It consists of two electrodes, an inner rod and an outer concentric shell with radii $R_1$ and $R_2$ forming an annular gap. Through the gap runs a gas volume flow *Q*. Particles are entrained from the inlet at $z=0$ in axial direction against gravity due to hydrodynamic drag from the fluid which exhibits a velocity profile $u_z(r)$ towards the outlet at $z=L$. Movement in radial direction is achieved by dielectrophoresis due to the inhomogenenous electric field ${\mathbf{E }}(r)$ (the spatial field inhomogeneity is $\left( {\mathbf{E }}\cdot \nabla \right) {\mathbf{E }}$) and classification is achieved due to the different dielectrophoretic mobility $\mu _\text {DEP}$ of particles of different size, shape, or material. The two Gaussian distributions drawn on top of the particle trajectory (red line) show the evolution of the probability distribution with time due to Brownian motion. (**b**) Top view of the classifier. (**c**) Exemplary random-walk particle trajectories including diffusion (black dashed) around the deterministic trajectory (red solid).

A schematic overview of a DEP particle classifier is given in Fig. 1. For better understanding, the DEP classifier concept will be introduced without accounting for additional in- and outlet flows as they may occur

1. when the aerosol is entering the classifier in a separated flow and
2. when the classified aerosol sample is separated into an additional outlet flow.

The model that we use for calculation includes the influence of gas flows at in- and outlet. It will be presented at the end of the section (cf. “Calculation model to account for in- and outlet flow” section).

The main part of the classifier is a cylindrical annulus with a central metal rod of outer radius $R_1$ and a concentric cylindrical metal shell of inner radius $R_2$. Both are connected to an ac voltage generator so that each part (rod and shell) forms one electrode (inner and outer electrode). The aerosol flows through the gap along the separation length *L*. In this idealized geometry we do not consider the flow distortion due to particle inlet and outlet, and we assume a one-way coupling, i.e., the gas flow *Q* is independent of the particles. We can therefore assume an undisturbed laminar annular flow profile (with velocity distribution $u_z(r)$). Here, it is assumed that particles are entering the classifier at $z = 0$ and $r = R_2$ and are carried along the axial direction against gravity ${\mathbf{g }}$ towards the outlet (particle collection section) at $z = L$.

Due to the concentric design the resultant electric field ${\mathbf{E }}$ is inhomogeneous with the maximum electric field strength at the surface of the inner electrode (see left inset in Fig. 1a containing $({\mathbf{E}}\cdot \nabla ){\mathbf{E}}$). This will motivate a (solely) radial DEP particle motion towards the inner electrode. A classification can be achieved because particles of different size, shape, and material will experience a different dielectrophoretic and drag force and will therefore move slower or faster towards the inner electrode.

In this paper we assume only spherical particles which will be classified by diameter. Due to Brownian motion the particles will not strictly follow the deterministic particle trajectory (depicted by the red line in Fig. 1a, c) but exhibit a diffusive broadening of their trajectories. Therefore, they will arrive at $x=L$ with a Gaussian distribution in *z*-direction and standard deviation $\sigma _z$ (see right inset in Fig. 1a). Figure 1c contains a schematic representation of the random walk which causes the Gaussian distribution around the ideal (red) trajectory of three exemplary particles. Figure 1b shows the proposed classifier from top view for completion. The system is axisymmetric and therefore given in two-dimensional cylindrical coordinates (*r*, *z*).

### Particle trajectory

The full differential equation describing the position ${\mathbf{x}}=(x_r,x_z)^{\mathsf{T}}$ of a *diffusive* particle with mass $m_\text {P}$ in the stationary electric and fluid field reads

1 $$\begin{aligned} m_\text {P} \frac{\partial ^2{\mathbf{x}}}{\partial t^2} = -{\mathbf{F}}_\text {D} - {\mathbf{F}}_\text {B} + {\mathbf{F}}_\text {EK} + {\mathbf{F}}_\text {DEP} + {\mathbf{F}}_\text {BM}. \end{aligned}$$

The left side of Eq. (1) represents the inertia force, ${\mathbf{F}}_\text {D}$ is the drag force, ${\mathbf{F}}_\text {B}$ is the buoyancy force, ${\mathbf{F}}_\text {DEP}$ is the DEP force, ${\mathbf{F}}_\text {BM}$ is a random force accounting for Brownian motion, and ${\mathbf{F}}_\text {EK}$ accounts for electrokinetic forces (such as electrophoresis or electroosmosis). The buoyancy force is ${\mathbf{F}}_\text {B} = V_{{\text {P}}} (\rho _{{\text {P}}}-\rho _{{\text {F}}}) {g}$, with the particle volume $V_{{\text {P}}}$, the densities of the particle and the ambient fluid $\rho _{{\text {P}}}$ and $\rho _{{\text {F}}}$ and the gravity constant ${g}={9.81}\,\hbox {ms}^{-2}$. The electrokinetic forces ${\mathbf{F}}_\text {EK}$ always depend on the direction of the field. The electric field frequency is chosen to be so high that spatial fluctuations due to electrokinetic (EK) movement are negligible compared to the spatial field change; hence, we can safely assume the time-averaged ${\mathbf{F}}_\text {EK}$ to be 0.

As stated above, two methods have been used in this study to account for particle diffusion. In the complex model, we use the Langevin approach^25^, where ${\mathbf{F}}_\text {BM}$ is a random force which changes direction and magnitude in each integration time-step of Eq. (1). Then, a vast number of trajectories have to be calculated to obtain a meaningful distribution of particle end positions. In our second approach to calculate diffusion, ${\mathbf{F}}_\text {BM}$ is set to zero and only the idealized trajectories are calculated for particle diameters of interest. We then account for Brownian motion by calculating Gaussian probability distributions of particles in *z*-direction along the idealized mean trajectories. Both methods have been compared against each other (see Results section, “Results and discussion” section) with very good agreement.

### Drag force

The drag force is calculated under the assumption of spherical particles according to Stokes law^8^,

2 $$\begin{aligned} {\mathbf{F}}_\text {D} = f \left( \frac{\partial {\mathbf{x}}}{\partial t} - {\mathbf{u}}_\text {F}\right) , \end{aligned}$$

with the fluid velocity ${\mathbf{u}}_\text {F} = (0,u_z)^{\mathsf{T}}$ and the friction coefficient $f = 3\pi \mu _\text {F}d_\text {P}/C$. Here, $\mu _\text {F}$ is the fluid (gas) viscosity, $d_\text {P}$ the particle diameter, and *C* the well-known Cunningham slip correction factor^8^, which has been calculated with the experimental constants $A_i$ from Davies^26^.

### DEP force

The DEP force on a spherical particle with diameter $d_\text {P}$ in an inhomogeneous electric field ${\mathbf{E}}$ can be calculated using the point-dipole approximation^27^:

3 $$\begin{aligned} {\mathbf{F}}_\text {DEP} = \frac{1}{2}\pi d_\text {P}^3 \varepsilon _0\varepsilon _\text {F}\text {Re}\left[ {\tilde{f}}_\text {CM} (\omega )\right] \left( {\mathbf{E}}\cdot \nabla \right) {\mathbf{E}}. \end{aligned}$$

Here, $\varepsilon _0 = {8.854 \times 10^{-12}}\,{\hbox {Fm}^{-1}}$ is the permittivity of free space, $\varepsilon _\text {F}$ the permittivity of the fluid (gas), which is approximately 1 for dry gases, $\text {Re}\left[ {\tilde{f}}_\text {CM}(\omega )\right]$ the real part of the Clausius–Mossotti function, and $\omega$ the angular frequency of the applied field. The Clausius–Mossotti function follows from the solution of the Laplace equation at the surface of a perfectly spherical particle and is given by:

4 $$\begin{aligned} {\tilde{f}}_\text {CM}(\omega ) = \frac{{\tilde{\varepsilon }}_{{\text {P}}}-{\tilde{\varepsilon }}_{{\text {F}}}}{{\tilde{\varepsilon }} _{{\text {P}}}+2 {\tilde{\varepsilon }}_{{\text {F}}}}, \qquad {\tilde{\varepsilon }}(\omega ) = \varepsilon + \frac{\sigma }{j \omega } . \end{aligned}$$

The complex permittivity ${\tilde{\varepsilon }}$ of a medium (subscripts P or F for particle or fluid, resp.) is dependent on its permittivity $\varepsilon$ and conductivity $\sigma$ and the angular frequency of the applied electric field $\omega$. The imaginary number is denoted by *j*. $\text {Re}\left[ {\tilde{f}}_\text {CM}(\omega )\right]$ describes the relative polarizability of the particle in the surrounding medium and, since all other variables in Eq. (3) are positive, decides whether a particle moves along ($\text {Re}\left[ {\tilde{f}}_\text {CM}(\omega )\right] > 0$, positive DEP) or against the gradient ($\text {Re}\left[ {\tilde{f}}_\text {CM}(\omega )\right] < 0$, negative DEP). In gases, this function is virtually independent of $\omega$ and is either 0 for non-conductive particles or 1 for conductive particles or materials with a very high permittivity ($\varepsilon _\text {P} \ge 1000$)^21^. If the function is 0, then the particle exhibits no DEP force. In this study we assume that $\text {Re}\left[ {\tilde{f}}_\text {CM}(\omega )\right] = 1$. The method proposed here is also applicable for non-conductive particle if humid air is used as a carrier gas, because this effectively makes small particles conductive and gives them a positive CM factor^20^.

### Brownian motion

Two different approaches have been used to account for Brownian motion and have been verified against each other in order to make sure that the particle diffusion has been implemented correctly. The first, more complex approach is based on the Langevin equation ^25^. In this case, we model the particle trajectory (Eq. (1)) massless and hence neglect inertia (the left side of Eq. (1) is 0). In a simple first-order forward time-stepping scheme we obtain as a particle position^25^

5a $$\begin{aligned} {\mathbf{x}}_{i+1}= {\mathbf{x}}_i + f^{-1}\left( {\mathbf{F}}_\text {DEP}+ \sqrt{\frac{24k_\text {B}Tf}{\Delta t}}{} {\mathbf{r}}_i\right) \Delta t, \end{aligned}$$

5b $$\begin{aligned} t_i= i \Delta t. \end{aligned}$$

Here, both ${\mathbf{r}}_i$ and ${\mathbf{F}}_\text {DEP}$ are given in (*x*, *y*, *z*)-coordinates and ${\mathbf{r}}_i$ is a 3d random number vector with all 3 components containing random numbers with zero mean and a variance of 1/12 and *i* is the current time step. To obtain a probability density of the particle’s position normal to the ideal trajectory it is necessary to calculate a vast number of trajectories using Eq. (5a) which, employing a first-order time-stepping scheme, may take up a lot of computational time.

**Figure 2:**
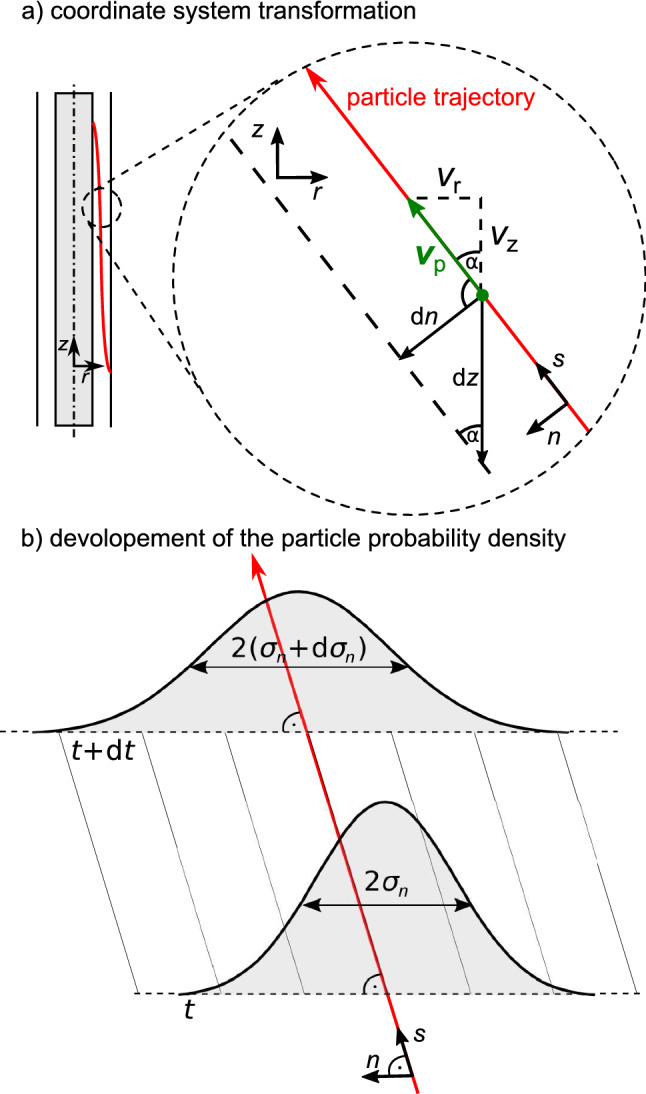
(**a**) Derivation of mean square displacement in *z*-direction. The global coordinate system is (*r*, *z*), the coordinate system (*s*, *n*) is in direction of the particle trajectory *s* and normal to it *n*. The particle’s velocity is ${\mathbf{v}}_\text {P} = (v_r,v_z)^{\mathsf{T}}$. The angle $\alpha$ is between the direction of *s* and *z*. (**b**) Development of the probability density of the particle’s position normal to the ideal trajectory (red arrow) during an infinite time step $\text {d}t$.

In a second approach we therefore calculated the mean square displacement in *z*-direction due to Brownian motion using geometrical considerations (see Fig. 2). We define a new coordinate system (*s*, *n*) where *s* points in the direction of the particle’s trajectory and *n* is the normal component to the trajectory. The origin of the coordinate system lies at the current position of the particle ${\mathbf{x}}$. We neglect diffusion in *s*-direction as it does not alter the position where the particle exits the classifier. The only impact of diffusion in *s*-direction is a small change in time when the particle reaches the exit. The differential of the mean square displacement in *n* direction reads

6 $$\begin{aligned} \text {d}\sigma _n^2=2D\text {d}t. \end{aligned}$$

Using the geometrical relations from Fig. 2 we can link the mean square displacement in *z*-direction to the displacement in *n*-direction and the velocity ${\mathbf{v}}_P=(v_r,v_z)^{\mathsf{T}}$ of the particle:

7 $$\begin{aligned} \text {d}\sigma _z^2 = \frac{1}{\sin ^2\alpha } \text {d}\sigma _n^2 = \left( \frac{\sqrt{v_r^2+v_z^2}}{v_r}\right) ^2 2 D \text {d}t. \end{aligned}$$

We assume that the particles always move at terminal velocity, thus $v_r$, $v_z$ and $\text {d}\sigma _z(r)$ are only dependent on the radial coordinate *r* and independent of *z*. Consequently, $v_r$ is defined by DEP and $v_z$ by the fluid flow. We can integrate Eq. (7) to find $\sigma _z$:

8 $$\begin{aligned} \sigma _z(r=r(t))=\sqrt{2D \int \limits _{0}^{t}\left( \frac{\sqrt{v_r^2+v_z^2}}{v_r}\right) ^2 \text {d}t^*}. \end{aligned}$$

It is worth mentioning that this expression looses accuracy with increasing particle distribution because in this calculation particles of the same size are treated as if they had the same residence time. In reality particles that diffuse from the average trajectory towards the outer electrode have a longer residence time than particles that diffuse from the average trajectory towards the inner electrode. That results in a slightly asymmetric distribution. Nevertheless, the comparison with Langevin trajectories (“Particle probability distribution due to Brownian motion” section) shows that the error is negligible.

### Flow field and electric field

The flow through the gap between the inner and outer electrode from the ideal classifier (Fig. 1a) is one-dimensional in *z*-direction and only a function of *r*. The velocity profile of a laminar flow through such an annulus with inner radius $R_1$, outer radius $R_2$ at gas flow rate $Q_t$ can be calculated by ^28^,

9 $$\begin{aligned} u_z(r) = \frac{2Q_t\left[ 1-\left( r/R_2\right) ^2-\frac{1-\kappa ^2}{\ln (1/\kappa )} \ln (R_2/r)\right] }{\pi \left( R_2^2-R_1^2\right) \left[ \frac{1-\kappa ^4}{1-\kappa ^2}-\frac{1-\kappa ^2}{\ln {1/\kappa }}\right] }, \end{aligned}$$

with the radius ratio $\kappa = R_1/R_2$.

The electric field can be calculated through the electrostatic potential $\Phi$ which is for such a concentric electrode arrangement given by^29^

10 $$\begin{aligned} \Phi (r)=U_0-\frac{U_0}{\ln (R_2/R_1)}\ln {\frac{r}{R_1}}, \end{aligned}$$

with the voltage applied across the gap $U_0$. Since $\Phi$ is only dependent on *r* (one variable), the resultant electric field ${\mathbf{E}}(r)=(E(r),0)^{\mathsf{T}}$ only depends on *r*. It is pointing towards (or away from) the central axis of the inner electrode and is parallel to the (*r*, *z*)-plane. The electric field *E*(*r*) and $({\mathbf{E }}\cdot \nabla ){\mathbf{E }}$ are given by:

11 $$\begin{aligned} E(r)= - \frac{\partial \Phi }{\partial r}=-\frac{U_0}{r\cdot \ln (R_2/R_1)}, \end{aligned}$$

12 $$\begin{aligned} ({\mathbf{E }}\cdot \nabla ){\mathbf{E }}=  \left( -\frac{\partial \Phi }{\partial r}\cdot \frac{\partial }{\partial r}\right) \left( -\frac{\partial \Phi }{\partial r}\right) = -\frac{U_0^2}{r^3\ln ^2(R_2/R_1)} . \end{aligned}$$

### Calculation model to account for in- and outlet flow

The DEP particle classifier concept presented above assumes a constant flow profile along the whole classifier and thereby neglects the gas flow distortion that is caused by the in- and outlet flow. To calculate classifier resolution and yield, we use a more complex model that includes in- and outlet flow.

**Figure 3:**
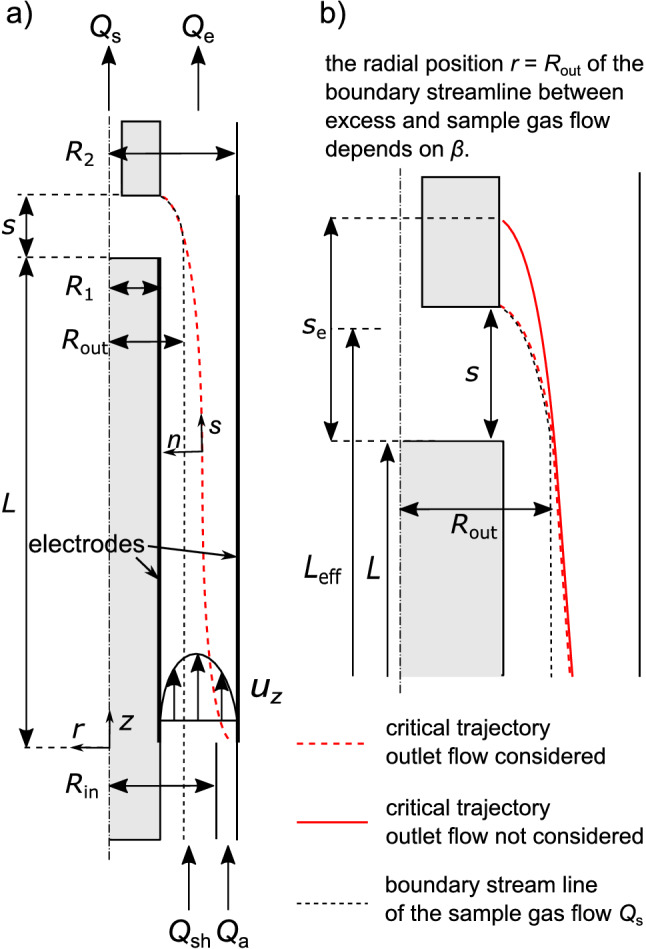
(**a**) Schematic representation of the classifier model around the symmetry axis which takes the incoming aerosol $Q_\text {a}$ and sheath gas flow $Q_\text {sh}$ and the outgoing sample $Q_\text {s}$ and excess gas flow $Q_\text {e}$ into account. The critical stream line that separates $Q_\text {s}$ from $Q_\text {e}$ (black dashed line) and a critical particle trajectory that barely follows $Q_\text {s}$ (red dashed line) are shown. (**b**) The critical particle trajectories that enters the outlet slit when the outlet drag force due to the sample gas flow is considered (red dashed line). When the drag force is not considered the same particle hits the inner electrode later (red line). Instead of implementing the drag force into the (simplified) particle trajectory calculation the effect of $Q_\text {s}$ is here compensated by extending the outlet slit from *s* to $s_\text {e}$ so that particles on the critical trajectory enter the outlet slit.

When looking at DMA, modelling the influence of gas flow is often considered in a simplified manner ^30–32^: It is assumed that the flow velocity profile in the gap between the electrodes is fully developed from the in- to the outlet of the classifier. The inlet is considered to be at the axial position $z=0$ between the radii $R_{{\text {in}}}$ and $R_2$ and the outlet at $z=L$ between $R_1$ and $R_{{\text {out}}}$ (all four radii are shown for a DEP classifier in Fig. 3a) whereas in- and outlet in the *actual* classifier are most of the time axial slits at the outer and the inner electrode. These radial widths of the (virtual) aerosol in- and sample outlet (set by $R_{{\text {in}}}$ and $R_{{\text {out}}}$) are determined by the gas flow ratio $\beta = (Q_{{\text {a}}}+Q_{{\text {s}}})/(Q_{{\text {sh}}}+Q_{{\text {e}}})$ (with $Q_{{\text {a}}}=Q_{{\text {s}}}$ and $Q_{{\text {s}}}=Q_{{\text {e}}}$). The flow ratio defines therefore the throughput at constant gas velocity. The radial positions $R_{{\text {in}}}$ and $R_{{\text {out}}}$ correspond to the boundary gas stream lines at in- and outlet assuming a developed flow profile $u_z(r)$ (cf. Eq. (9)):

13 $$\begin{aligned} \frac{\beta }{1-\beta }=  \frac{\pi \int _{R_\text {in}}^{R_2}r u_z(r) \text {d}r}{\pi \int _{R_1}^{R_\text {in}}r u_z(r) \text {d}r}, \end{aligned}$$

14 $$\begin{aligned} \frac{1-\beta }{\beta }=  \frac{\pi \int _{R_\text {out}}^{R_2}r u_z(r) \text {d}r}{\pi \int _{R_1}^{R_\text {out}}r u_z(r) \text {d}r} . \end{aligned}$$

In our classifier model (Fig. 3) we used this method only for the inlet and assumed that the particles are homogeneously distributed in the aerosol flow $Q_{{\text {a}}}$. Since the standard deviation of the spatial particle distribution due to Brownian motion can only be calculated in axial but not in radial direction (see “Brownian motion” section) a radially stretched outlet slit is unsuited for calculation of a transfer function. Therefore, in our simulations we deviate from the established DMA methodology and leave the outlet slit at the inner electrode $r = R_1$.

The method that we use to calculate the standard deviation of the spatial particle distribution $\sigma _z$ requires that the particle velocity in radial direction $v_r$ is independent of axial position *z* (see “Brownian motion” section). This is not given with a sample flow $Q_{{\text {s}}}$ because it leads to an additional acceleration of the particles at the outlet. To compensate for the influence of the flow field at the outlet while keeping the analytical expressions for the gas flow profile and the DEP force untouched we assumed a virtually *extended* outlet slit, which is longer than the actual slit. Extending the outlet slit means that particles have additional time so that the DEP force (instead of the gas flow towards the outlet slit) can pull particles into the sample outlet.

The idea may seem constructed but gives us an elegant way to keep the simple calculation algorithm for the mean particle trajectory and its standard deviation. The extended outlet slit width $s_{{\text {e}}}$ can be determined by calculating the time that a particle needs to travel from $R_{{\text {out}}}$ to the inner electrode $R_1$ and the axial distance that the particle migrates due to the gas flow during this time,

15 $$\begin{aligned} s_{{\text {e}}} = \int \limits _{t(r = R_{{\text {out}}})}^{t(r = R_1)} u_z(r(t))\text {d}t , \end{aligned}$$

with $u_z(r)$ from Eq. (9). The radial coordinate *r*(*t*) is derived from $\text {d}r/\text {d}t = v_{{\text {DEP}}}(r)$ with the final DEP migration velocity,

16 $$\begin{aligned} \frac{\text {d}r}{\text {d}t} = v_{{\text {DEP}}}(r) = -\frac{{d_{{\text {P}}}}^2 \varepsilon _0 \varepsilon _{{\text {F}}} \text {Re}\left[ {\tilde{f}}_\text {CM}(\omega )\right] C}{6 \mu _{{\text {F}}}} \left( \frac{U_0}{\ln ({R_2}/{R_1})}\right) ^2 \frac{1}{r^3} = C_{{\text {vr}}} \frac{1}{r^3}. \end{aligned}$$

For simplification we introduce the *r*-independent part $C_{{\text {vr}}}$, which is dependent on the particle diameter. With the boundary condition $r(t={0}\,\hbox {s})=R_2$ it follows,

17 $$\begin{aligned} t(r) = \frac{1}{4 C_{{\text {vr}}}} \left( r^4 - R_2^4\right) \end{aligned}$$

which can be rearranged to

18 $$\begin{aligned} r(t) = \left( 4 C_{{\text {vr}}} t + R_2^4 \right) ^{1/4}. \end{aligned}$$

We define an effective classifier length which ends in the middle of the extended outlet slit $L_{{\text {eff}}} = L + s_{{\text {e}}}/2$. The particle with the average terminal position $L_{{\text {eff}}}$ has the diameter that the classifier is calibrated for.

To justify our approach, we recomputed the transfer function from Hagwood *et al.*^30^, the results can be found in the supplementary information. Our results are in excellent agreement with theirs (which were calculated using a Monte Carlo approach and using the analytical solution of Stolzenburg^33^), which we cannot use in case of a DEP-driven classification.

Further, this model is only valid when particle inertia is negligible so that the particle is ideally following the gas flow. Relaxation times $t_{{\text {r}}} = ({\rho _{{\text {P}}} d_{{\text {P}}}})/({18\, \upmu _{{\text {F}}}})$ of particles with diameters of ${10}\,\upmu \hbox {m}$, ${20}\,\upmu \hbox {m}$, and ${40}\,\upmu \hbox {m}$ and density of ${2{,}000}\,\hbox {kg m}^{-3}$ are 0.006 s, 0.024 s, and 0.096 s. The time for a particle to pass the slit should be at least one order of magnitude higher. In our calculations this was the case for particles smaller ${10}\,\upmu \hbox {m}$.

## Methodology

The results for particle size (“Particle size range” section) and particle probability distribution (“Particle probability distribution due to Brownian motion” section) were calculated with $\beta =0$ ($Q_{{\text {a}}}=0$ and $Q_{{\text {s}}}=0$) using Eq. (1) and ${\mathbf{F}}_\text {BM} = 0$ while particle diffusion was calculated with Eq. (8) (the simple mean-square-displacement model). We additionally made comparison to validate that Eq. (8) is correct using Eq. (5a) (the Langevin approach), which is indicated at the appropriate figure. Results for the resolution and yield were obtained considering aerosol inlet and sample gas flows, $Q_{{\text {a}}}$ and $Q_{{\text {s}}}$. We chose four exemplary values for $\beta$ between 0.01 and 0.1.

**Figure 4:**
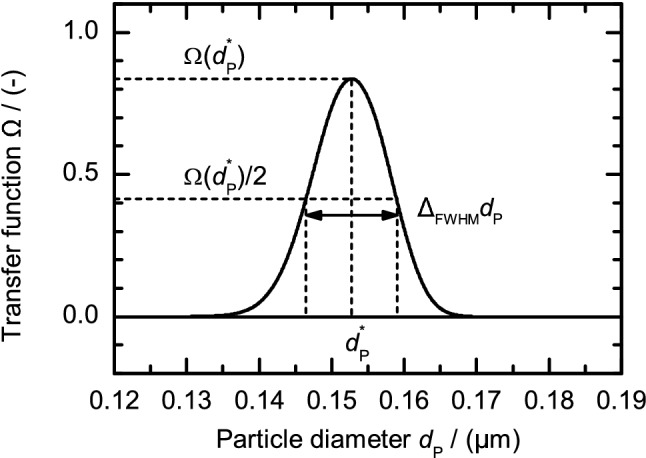
Exemplary transfer function $\Omega (d_\text {P})$. The transfer function is the fraction of particles with size $d_\text {P}$ that have been transferred from the aerosol inlet flow $Q_\text {a}$ to the sample gas flow $Q_\text {s}$. In an ideal case it should be 1 for $d_\text {P}=d_\text {P}^*$ and 0 for all other $d_\text {P}$, but it is broadened due gas flow ratio at in- and outlet $\beta$ and particle diffusion.

The general methodology is to calibrate the separator design for a specific target particle diameter $d_\text {P}^*$. The target particle diameter is the diameter of non-diffusive particles that reach $r = R_1$ at $z = L$ ($\beta =0$) or $z = L_{{\text {eff}}}$ ($\beta >0$). Due to diffusion and an outlet slit with finite dimensions a fraction of smaller and bigger particles than $d_\text {P}^*$ will exit the classifier with the sample gas flow. Also, if the probability distribution is broader than the outlet slit width, not all particles that should be classified (i.e. having a diameter $d_\text {P}^*$) will exit the classifier with the sample gas flow. These two effects are quantified by the resolution that we introduce as

19 $$\begin{aligned} R = \frac{d_\text {P}^*}{\Delta _\text {FWHM}d_\text {P}} \end{aligned}$$

with $\Delta _\text {FWHM}d_\text {P}$ the full width at half maximum (FWHM) of the particle size distribution and by the transfer function $\Omega (d_\text {P})$ which gives the fraction of particles with diameter $d_\text {P}$ that are transferred from the inlet aerosol gas flow $Q_\text {a}$ to the outlet sample gas flow $Q_\text {s}$ (cf. Fig. 4). In an ideal case, $\Omega (d_\text {P}=d_\text {P}^*)=1$ and $\Omega (d_\text {P}\ne d_\text {P}^*)=0$, which would yield an infinite resolution $R = \infty$. Due to diffusion the transfer function shows a Gaussian distribution and $\Omega (d_\text {P}\ne d_\text {P}^*) \ne 0$. Also, when the probability distribution of particle positions in *z*-direction at the outlet is wider than the in “Calculation model to account for in- and outlet flow” section introduced extended outlet slit, the value of $\Omega (d_\text {P} = d_\text {P}^*)$ is lower than one.

All calculations have been performed using an outer diameter of $R_2 = {10}\,\hbox {mm}$ and an inner diameter of $R_1 = {7}\,\hbox {mm}$. The length of the classifier has been varied between 6 and 600 cm. The applied potential was varied between 0.33 and 76.6 kV which result in maximum field strength at the inner electrode of $E(r=R_1)={133}\,\hbox {kV m}^{-1}$ and $E(R_1)={30.5}\,\hbox {MV m}^{-1}$. It is limited by the dielectric strength of air and is the maximum applied field strength that can be applied without risking breakdown. According to Flagan^34^ this is, for two concentric cylinder electrodes, one third of the field strength given by Paschen’s Law for concentric DMA. We did not apply Paschen’s Law but used one third of the experimental values from Cohen^35^ at pressures between $p = {1}\,\hbox {bar}$ (lower end) and $p = {60}\,\hbox {bar}$ (upper end) instead. Below the breakdown voltage the electric conductivity of air is so low that temperature rise due to Joule heating is negligible. The mean gas velocity of the annular flow ($u_{{\text {gas}}} = \overline{u_z}$) was varied between $u_\text {gas} = {0.1}\,\hbox {cm s}^{-1}$ and $u_\text {gas} = {514}\,\hbox {cm s}^{-1}$. The upper value of the gas velocity is the highest attainable velocity that would still yield a laminar flow at $p = {1}\,\hbox {bar}$ at the chosen values of $R_1$ and $R_2$. The real outlet slit width for was set to $s={1}\,\hbox {mm}$

The relative permittivity of air has been approximated as $\varepsilon _\text {r,air} = 1$, the dynamic viscosity as $\mu ={18.6}\,\upmu \hbox {Pas}$ for a gas temperature of $T = {293}\,\hbox {K}$. The mean free path of air has been calculated using^8^ $\lambda = RT/(\sqrt{2}\pi p N_\text {A}d_\text {m}^2)$ with the universal gas constant $R={8.314}\,\hbox {Jmol}^{-1}\,\hbox {K}^{-1}$, the Avogadro constant $N_\text {A}={6.022\times 10^{23}}\,\hbox {mol}^{-1}$ and the mean collision diameter of air molecules $d_\text {m} = {3.7}$ Å. The density of air has been calculated using the ideal gas law, $\rho = p/(R_\text {s}T)$ with the specific gas constant of air, $R_\text {s} = {287.06}\,\hbox {J kg}^{-1}\,\hbox {K}^{-1}$. The particle’s density was assumed to be $\rho _\text {P} = {2{,}000}\,\hbox {kg m}^{-3}$.

Equation (5a) has been solved using a for-loop realized in MATLAB to calculate the particle trajectory in time steps of $\Delta t={1}\,{\upmu \hbox {s}}$ which is sufficiently small that particle trajectories and particle distributions remained unaffected by further time-step refinement. The differential equation (1) together with standard deviation (8) have been solved using MATLAB’s ODE45 suite using error tolerances of $10^{-8}$ (absolute) and $10^{-5}$ (relative).

## Results and discussion

### Particle size range

**Figure 5:**
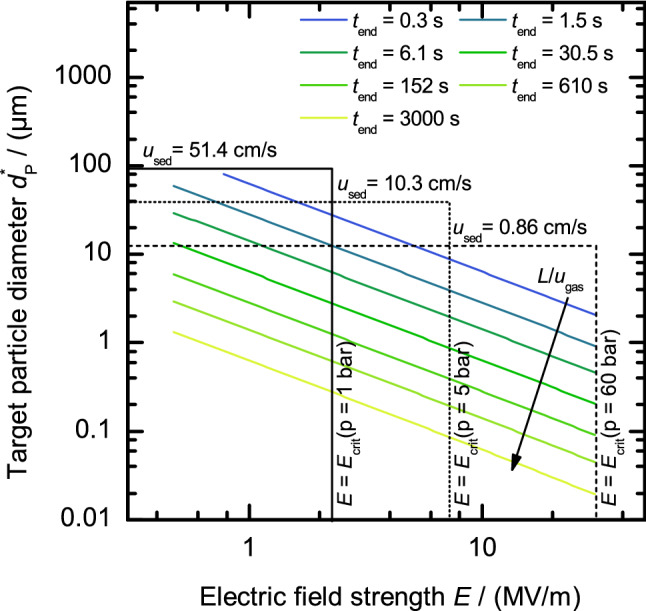
Numerically calculated target particle diameter $d_{{\text {P}}}^*$ as a function of the electric field strength *E* for varied residence times $t_{{\text {end}}}$. The target particle diameter changes proportional to $t_{{\text {end}}}^{-0.5} \propto (u_{{\text {gas}}}/L)^{0.5}$ (arrow). The solid (${1}\,\hbox {bar}$), dotted (${5}\,\hbox {bar}$), and dashed (${60}\,\hbox {bar}$) lines indicate the maximum allowed electric field strength at a specific pressure (higher *E* could lead to breakdown) and the maximum particle diameter defined by sedimentation; larger particles would require higher mean gas velocities ($u_{{\text {gas}}} \ge 10 \, u_{{\text {sed}}}$), which in turn would lead to non-laminar flow.

The particle movement depends on the DEP force in radial direction and the sum of drag force exerted by the gas and the gravitational body-force in axial direction. Since the distance between the electrodes is fixed at 3 mm, only the electric field gradient and the particle diameter influence the residence time $t_{{\text {{end}}}}$. As residence time we define the time that a particle needs to travel from the outer electrode at $R_2$ (the particle starting point) towards the inner electrode at $R_1$ (where the outlet slit is located), i.e., the time a particle needs to traverse across the annular gap due to DEP. During that time the particle moves in axial direction depending on the gas velocity and its sedimentation velocity. For a given electric field strength *E* the target particle diameter $d_{{\text {P}}}^*$ (diameter of the particle that hits the inner electrode at length *L* ($\beta =0$)) is therefore determined by the classifier length *L* and the mean gas velocity $u_{{\text {gas}}}$. Because the flow direction of the classifier was chosen upwards, bigger particles travel slower in axial direction but faster in radial direction due to the cubic dependence of the DEP force on the particle diameter. As a consequence particles will hit the inner electrode at different positions depending on their size. The smaller the particle, the longer the axial distance it travels, because it has a longer residence time (lower radial velocity) and a higher axial velocity. Figure 5 shows the target particle diameter $d_{{\text {P}}}^*$ as a function of the electric field strength *E* for several particle residence times between 0.3 s and 3,000 s. Because the target particle diameter depends on *L* and $u_\text {gas}$, the *target* particle residence time $t_{{\text {end}}}^*$ is directly dependent on the ratio of $L/u_{{\text {gas}}}$. If the sedimentation velocity is much smaller than the mean gas velocity, it follows

$$\begin{aligned} t_{{\text {end}}}^* = 1.017 \frac{L}{u_{{\text {gas}}}} . \end{aligned}$$

Figure 5 shows that, under the given conditions, the target particle diameter follows

20 $$\begin{aligned} d_{{\text {P}}}^*= {34.9}\,{\hbox {Vs}^{0.5}}(L/u_{{\text {gas}}})^{-0.5} E^{-1} \end{aligned}$$

with a coefficient of determination $R^2$ higher than 0.99. The factor ${34.9}\,{\hbox {Vs} ^{0.5}}$ accounts for the classifier geometry and the gas and particle properties. We generalize that finding for cylindrical classifiers: When the target particles diameter is one order of magnitude (OM) larger than the mean free path of the gas molecules, the target particle diameter is proportional to

21 $$\begin{aligned} d_{{\text {P}}}^*\propto t_{{\text {end}}}^{-0.5} E^{-1} \propto (L/u_{{\text {gas}}})^{-0.5} E^{-1} . \end{aligned}$$

Two limitations need to be considered:

1. The vertical lines in Fig. 5 show the maximum electrical field strengths that cannot be exceeded without risking gas ionization at pressures of ${1}\,\hbox {bar}$ (solid line), ${5}\,\hbox {bar}$ (dotted line), and ${60}\,\hbox {bar}$ (dashed line). This limit increases with pressure as discussed in “Methodology” section. The maximum usable electric field strengths depending on the pressure are: $E(p={1}\,\hbox {bar})={1.2}\,\hbox {MV m}^{-1}$, $E(p={5}\,\hbox {bar})={5.4}\,\hbox {MV m}^{-1}$, and $E(p={60}\,\hbox {bar})={30.5}\,\hbox {MV m}^{-1}$.
2. The horizontal lines in Fig. 5 represent the particle diameter that has a sedimentation velocity of 10% of the mean gas velocity and thus gives the upper limit for classification. The classification of bigger particles is possible but their trajectories change entirely compared to particles having a sedimentation velocity below 10% of $u_{{\text {gas}}}$: The residence time would increase significantly so that particle diffusion has a much higher impact on particle classification. An increase in target particle diameter further without exceeding the 10% limitation requires to increase the mean gas velocity. At this point it must be considered that the flow changes from laminar to the transition regime at $Re = 2{,}000$. For the geometry chosen here and pressures of 1 bar, 5 bar, and 60 bar, this results in maximum mean gas velocities of $u_{{\text {gas}}} = {514}\,\hbox {cm s}^{-1}$, $u_{{\text {gas}}} = {103}\,\hbox {cm s}^{-1}$, and $u_{{\text {gas}}} = {8.6}\,\hbox {cm s}^{-1}$, respectively.

It is worth mentioning that the maximum residence time of 3,000 s is probably not practicable without having particle interaction, i.e. agglomeration. However, because this is a theoretical study, with the purpose to show dependencies and possibilities of DEP classification, it was taken as the limit. Experimental investigations need to follow. If the working pressure is 1 bar and if a maximum residence time of $t_{{\text {end}}} = {3{,}000}\,\hbox {s}$ is acceptable, the classifiable particle size ranges from approximately 300 nm to about ${90}\,\upmu \hbox {m}$ (restricted by dotted lines). For working pressures of 5 bar and 60 bar particles with diameters from approximately 80 nm to ${40}\,\upmu \hbox {m}$ and 20 nm to ${12}\,\upmu \hbox {m}$ (restricted by the dotted and dashed lines) can be classified.

### Particle probability distribution due to Brownian motion

The presented results alone say nothing about the selectivity of the classification process because they don’t include particle diffusion so far. In the following we will show how diffusion influences the classification result by approximating the maximum resolution and yield (maximum of the transfer function) that can be achieved by DEP classification depending on the particle size range. The results of this section were calculated with $\beta =0$ and the simple algorithm to calculate the diffusion (Eq. (7)).

**Figure 6:**
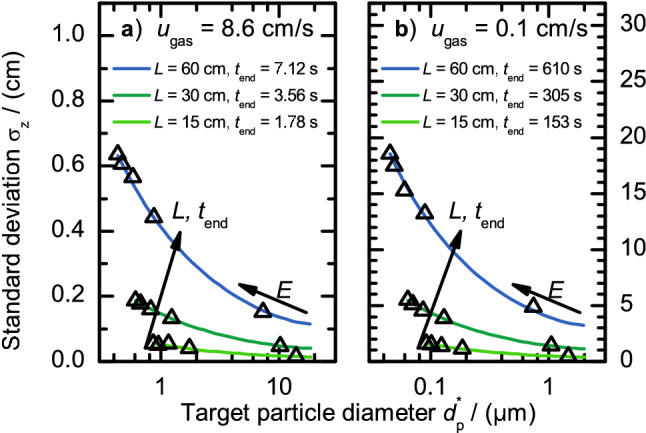
Standard deviation $\sigma _z$ as a function of target particle diameter $d_{{\text {P}}}^*$ for different classifier lengths *L*. The electric field strength *E* increases from right to left. The mean gas velocities are $u_{{\text {gas}}} = {8.6}\,\hbox {cm s}^{-1}$ (**a**) and $u_{{\text {gas}}} = {0.1}\,\hbox {cm s}^{-1}$ (**b**). The lines give the results calculated with the mean square displacement model (Eq. (8)). The triangles show results extracted from particle trajectories calculated through the Langevin model (Eq. (5a)).

As presented before the probability distribution of a particle‘s position around its average trajectory, which is the trajectory of a non-diffusing particle, can be assumed to be a Gaussian normal distribution. The normal distributions $\sigma _{z}$ of selected cases as a function of target particle diameter $d_{{\text {P}}}^*$ are presented in Fig. 6 for a variation of classifier lengths *L* and two gas velocities of $u_{{\text {gas}}} = {8.6}\,\hbox {cm s}^{-1}$ (a) and $u_{{\text {gas}}} = {0.1}\,\hbox {cm s}^{-1}$ (b). The electric field strength increases from right to left on each graph. The residence time along the lines is virtually constant but can be up to 11% longer than shown in the legend for the biggest particles (steep increase of the residence time as soon as the sedimentation velocity approaches 10% of the gas flow velocity). The analytical approach for the standard deviation (lines) matches very well with our additional test simulations using the Langevin approach (triangles). Each triangle shows the standard deviation of 1,000 Langevin particle trajectory calculations. The biggest relative difference is 8%; hence we can safely assume that the analytic approach gives good results for determining the particle probability distribution over the whole parameter range considered in this manuscript.

### Transfer function

Using the probability distribution and the average trajectory for each particle size and inlet position (weighted by the aerosol flow per cross section), a transfer function as described in the “Methodology” section can be determined. The size-dependent transfer function describes the probability that a particle is transferred from the inlet into the outlet sample flow. Two values characterize the transfer function and therefore the classification process:

1. Resolution: The narrower the transfer function, the higher is the size-selectivity of the classifier, which is described by the resolution (see Eq. (19)). The higher the resolution, the sharper the separation by particle size.
2. Maximum yield: The second size for describing the classification quality is the yield that the classifier achieves for the target particle diameter $\Omega (d_{{\text {P}}}^*)$. The yield is the probability that a particle with the target particle diameter $d_{{\text {P}}}$ is transferred from the inlet into the outlet sample flow.

**Figure 7:**
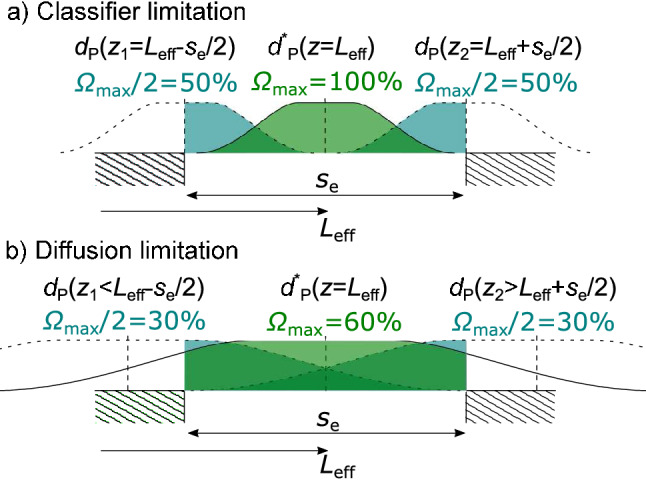
Limitation of the resolution *R* due to the classifier (process parameters and classifier geometry) (**a**) and due to particle diffusion (**b**). For both cases the particle probability distribution at the outlet and the respective percentage that hits the outlet slit $\Omega$ is shown for three different particle diameters that define the classifier resolution. The average terminal positions of the particle diameters that fulfill $\Omega = \Omega (d_{{\text {P}}}^*)/2=\Omega _{{\text {max}}}/2$ and thus determine the full width at half maximum of the transfer function are $z_1$ and $z_2$. Note that the particle probability distributions are shown schematically and do not illustrate the real quantitative distributions. The distributions are drawn flattened since a uniform (rectangular) distribution was assumed at the inlet.

The ratio of extended outlet slit width $s_{{\text {e}}}$ to the width of the spatial particle distribution of the target particle diameter in *z*-direction has a severe effect on both the resolution and the maximum yield as shown schematically in Fig. 7. Two cases of resolution limitation can be distinguished: *classifier limitation* and *diffusion limitation*. Keep in mind that the extended outlet slit width $s_{{\text {e}}}$ is an auxiliary value which does not reflect the physical classifier design but is used here to describe the influence of $\beta$. It is calculated by Eq. (15) using Eq. (14) to determine $R_{{\text {out}}}$.

If the particle distribution is small compared to the outlet slit width, *classifier limitation*, cf. Fig. 7a, the maximum yield is 100%. According to the definition of the resolution from Eq. (19) this case leads to the expression

22 $$\begin{aligned} R = \frac{d_{{\text {P}}}^*(z=L_{{\text {eff}}})}{d_{{\text {P}}}(z_1=L_{{\text {eff}}}-s_{{\text {e}}}/2) - d_{{\text {P}}}(z_2=L_{{\text {eff}}}+s_{{\text {e}}}/2)} . \end{aligned}$$

The particle sizes $d_{{\text {P}}}(z_1)$ and $d_{{\text {P}}}(z_2)$ are the two particles that fulfil $\Omega = \Omega (d_{{\text {P}}})/2$. They define the full width at half maximum of the transfer function. Their terminal positions are $z_1$ and $z_2$. With the proportionality of $d_{{\text {P}}} \propto L^{-0.5}$ from Eq. (21) it follows that

23 $$\begin{aligned} R = {\frac{L_{{\text {eff}}}^{-0.5}}{z_1^{-0.5}-z_2^{-0.5}}} \end{aligned}$$

and with $L_{{\text {eff}}}=L+s_{{\text {e}}}/2$, $z_1=L_{{\text {eff}}}-s_{{\text {e}}}/2 = L$, and $z_2=L_{{\text {eff}}}+s_{{\text {e}}}/2 = L+s_{{\text {e}}}$, the classifier limited resolution $R_{{\text {cl}}}$ is

24 $$\begin{aligned} R_{{\text {cl}}} = {\frac{(L+s_{{\text {e}}}/2)^{-0.5}}{L^{-0.5}-(L+s_{{\text {e}}})^{-0.5}}}. \end{aligned}$$

If the target particle distribution is of the same size or wider than the extended outlet slit width, the resolution does not follow Eq. (24) any longer, but is reduced by diffusion. As shown in Fig. 7b, the maximum yield $\Omega _{{\text {max}}}$ is then smaller than 100%. Consequently the full width at half maximum is not any longer determined by the extended outlet slit width, but by the positions $z_1 < L$ and $z_2>L+s_{{\text {e}}}$. Using Eq. (23) with $z_1 < L$ and $z_2>L+s_{{\text {e}}}$ shows that for diffusion limitation $R<R_{{\text {cl}}}$,

25 $$\begin{aligned} R = \frac{d_{{\text {P}}}^*(z=L_{{\text {eff}}})}{d_{{\text {P}}}(z_1) - d_{{\text {P}}}(z_2)} = \frac{L_{{\text {eff}}}^{-0.5}}{z_1^{-0.5}-z_2^{-0.5}} < \frac{L_{{\text {eff}}}^{-0.5}}{L^{-0.5}-(L+s_{{\text {e}}})^{-0.5}} = R_{{\text {cl}}}. \end{aligned}$$

It shows that diffusion-limited resolution is always smaller then the classifier-limited resolution $R_{{\text {cl}}}$. $R_{{\text {cl}}}$ depends on the classifier geometry, the flow ratio $\beta$, the flow velocity, and the applied electric field strentgh. This effect was described for DMAs in Ref.^36^. In the diffusion-limited case the resolution is further decreased by diffusional spreading with decreasing particle diameter. The higher the amount of in- and outlet flow, the lower the maximum achievable resolution.

It is obvious that, when the flow ratio $\beta$ and as a consequence the extended outlet slit width $s_{{\text {e}}}$ is reduced, resolution increases because the full width at half maximum or the distance between $z_1$ and $z_2$ decreases. But this increase in resolution is inevitably linked to a reduced yield.

#### Maximum achievable resolution

**Figure 8:**
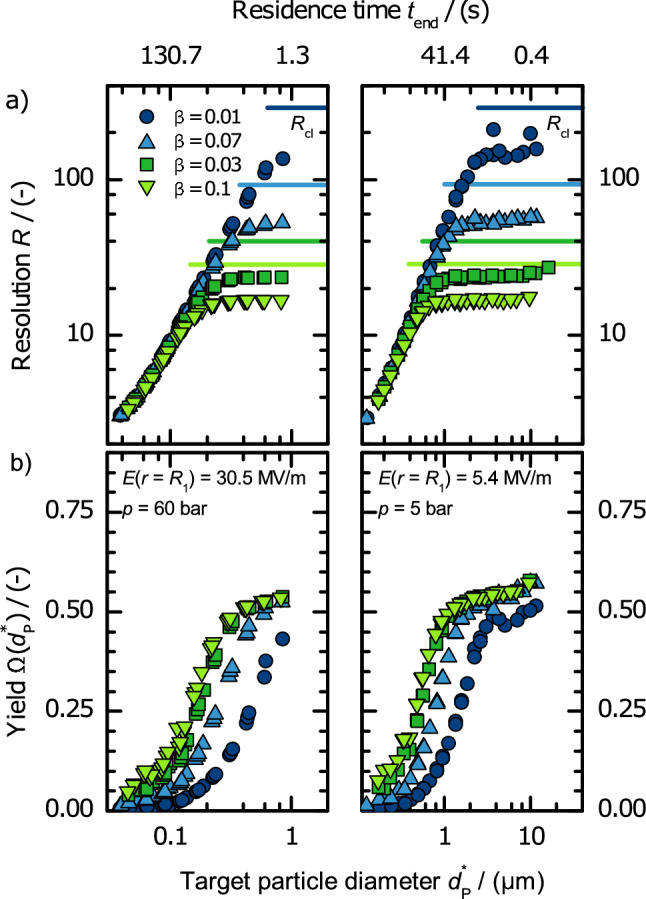
Resolution *R* (**a**) and yield $\Omega (d_{{\text {P}}}^*)$ (**b**) depending on the target particle diameter $d_{{\text {P}}}^*$ at pressures of 60 bar (left) and 5 bar (right) for different flow ratios $\beta$. The respective residence time is shown on the top axis. The solid lines show the classifier-limited resolution $R_{{\text {cl}}}$ (cf. Eq. (24)).

The highest resolution is achieved when the electric field strength is at the maximum value (defined by the dielectric strength), so that the dielectrophoretic force is maximized and the residence time (and thus the time available for spreading of the probability distribution due to diffusion) is minimized. Figure 8a shows the maximum achievable resolution depending on the particle diameter for different flow ratios $\beta$ and electric field strengths of ${30.5}\,\hbox {MV m}^{-1}$ and ${5.4}\,\hbox {MV m}^{-1}$. The two electric field strengths presented in this plot correspond to the maximum applicable (cf. “Methodology” section) at $p = {60}\,\hbox {bar}$ and $p = {5}\,\hbox {bar}$. The particle residence time correlates to the particle diameter and the electric field strength, $t_{{\text {end}}} = E^{-2} {d_{{\text {P}}}^*}^{-2} {1.2\times 10^{3}}\,{\hbox {V}^2\,\hbox {s}}$.

All graphs show the same behavior. Resolution increases with increasing particle diameter until a maximum is reached and resolution remains constant. This particle-size independent resolution is the classifier limitation. This maximum resolution is lower than the theoretical classifier-limited resolution $R_\text {cl}$ (Eq. (24)). Only for the largest particles (points on the right end of the graph), the resolution increases slightly again. The constant maximum of each graph depends on the flow ratio $\beta$. The higher the flow ratio (and thus the throughput) the lower the maximum resolution achieved. Resolution is limited to 160 for $\beta = 0.01$, 55 ($\beta = 0.03$), 24 ($\beta = 0.07$), and 17 ($\beta = 0.1$). Before this resolution maximum is reached, thus in the diffusion-limited case, the resolution is independent of $\beta$.

For small particles the resolution is determined by particle diffusion because the standard deviation in *z*-direction is broader than the extended outlet slit width ($s_{{\text {e}}}<\sigma _z$, cf. Fig. 7). With increasing particle size the particle distribution due to particle diffusion gets narrower whereas the extended outlet slit width remains the same. When the extended outlet slit width is about 4 times larger than the standard deviation, the classifier limitation is reached and resolution remains constant with increasing particle diameter.

In the diffusion-limited regime, the resolution is proportional to about ${d_{{\text {P}}}^*}^{1.3}$. This particle-size dependence is because small particles show larger residence time and more diffusional spreading. The dependency on the electric field strength is linear. When the resolution is classifier limited, the flow ratio $\beta$ is determining the maximum achievable resolution. The slight increase in resolution at highest particle diameter is because the residence time increases due to their increased sedimentation velocity of up to 10% of the average gas flow velocity. A few resolutions diverge from the trend of the others. These calculation artefacts occur due to small calculation inaccuracies. Especially for small $\beta$ values, slight inaccuracies of the particle trajectories can make a difference because the extended outlet slit width, where the particles are captured, is also small. Still the general trend is clearly evident.

For visualization: At a field strength of ${30.5}\,\hbox {MV m}^{-1}$ a resolution of about 10 is achieved for a target particle diameter of 100 nm. That means the particle diameters of about 95 nm and 105 nm are found with half the maximum yield in the sample flow. This resolution would be very attractive for some applications.

The resolution is always lower than the theoretically calculated classifier-limited resolution $R_{{\text {cl}}}$ (Eq. (24)). The classifier-limited resolution gives the maximum achievable resolution for the ideal case that all particles enter the classifier at the exact same position. In reality and also in our calculations, particles enter the classifier at different radial positions between $R_{{\text {in}}}$ and $R_{{\text {2}}}$ which results in lower resolutions.

#### Yield

Figure 8b shows the yield $\Omega (d_{{\text {P}}}^*)$ for the same cases as presented in Fig. 8a. The yield decreases with the target particle diameter following an s-shape. This is because the particle distribution of the target particle diameter is increasing with decreasing particle size and is broader than the extended outlet slit width. At particle diameters where the process changes from diffusion to classifier limitation the slope decreases and the yield approaches a maximum of 0.5 to 0.57. The yield is also increasing with increasing flow ratio $\beta$ (increasing throughput) because the extended outlet width is increasing with increasing $\beta$ and thereby a broader section of the particle distribution enters the sampling flow.

From our description of diffusion and classifier limitation it could be expected that the yield would approach 1. The reason for values below 1 is that the particles enter the classifier between $R_{{\text {in}}}$ and $R_2$. During classification these particles are spread due to their dielectrophoretic migration velocity to a radial section that corresponds to $f_{{\text {spread}}}$. The spreading factor is given by

26 $$\begin{aligned} f_{{\text {spread}}} = \frac{t_2}{t_1} , \end{aligned}$$

with $t_2$ being the time that a particle needs to travel in radial direction from $R_{{\text {2}}}$ to $R_{{\text {in}}}$ and $t_1$ the time that a particle needs to travel from $R_{{\text {out}}}$ to $R_{{\text {1}}}$. For the given geometry the spreading factor $f_{{\text {spread}}}$ is 1.9 for $\beta = 0.1$ and 2 for $\beta = 0.01$. This explains well why the yield is limited to about 50%. For higher yields the ratio between aerosol and sheath gas flow $\beta _{{\text {in}}}$ needs to be lower than the ratio between sampling and excess gas flow $\beta _{{\text {out}}}$. This does also explain that the resolution does not reach the derived classifier limitation $R_{{\text {cl}}}$, which is only achieved when $f_{{\text {spread}}} \le 1$.

Some calculation artefacts occur also for the yield. The explanation is the same as for the calculation artefacts present for the resolution, slight inaccuracies of the trajectory calculations.

### General remarks

All calculations are based on single particles. Particle interactions, which are increasingly occurring at higher particle concentrations and residence times, are neglected. The impact of particle interactions on the classification process are hard to predict by calculations and must be investigated experimentally. Barom *et al.*^21^ found a bimodal size distribution in their experiments on DEP fiber classification in air. They assumed that the smaller fibers stick to the bigger fibers and therefore both are found in the same sample. Further, they found that fibers deposit on the inner electrode at high fiber concentrations of ${2{,}900}\,\hbox {cm}^{-3}$ or larger. Nevertheless our results give a good impression of the principal possibilities of DEP classification under ideal conditions when no or few particle interaction occurs. To avoid accumulation, clustering, and sliding of particles at the inner electrode (which might lead to plugging the gap between the electrodes, increased electrical current due to conduction through particle agglomerates, and reduced resolution) two options can be considered. The first concept was applied by Baron *et al.*^21^, who used a thin oil film at the inner electrode to stop particles from re-dispersion and clustering. In this case the classification can only be operated as a batch process since particles need to be removed from the inner electrode at a certain particle loading. The second option is to add particle collectors (e.g. in form of a slits) at the inner electrode that collect particles that hit and slide down the inner electrode. This might allow a continuous operation. Simple discontinuous operation without oil film would also be possible to clean the classifier during the breaks. When considering higher particle loading the interaction of particle and fluid changes. The drag force becomes inversely proportional to the particle concentration which can be taken into account by using a drag force correction factor^37^.

A comparison of DEP classification to Differential Mobility Classification is hard to perform because classification in DMA devices occurs according to particle’s electrophoretic mobility which is, due to the particle charging process, not directly size-dependent but underlies stochastic variations. The resolution *R* of DEP classification is related to the dielectrophoretic mobility and is thus not comparable. The classifications yield $\Omega _{{\text {max}}}$ is given as a function of particle size for most DMA devices and can be compared. Two fundamental differences between DEP classification and DMA should be considered:

1. An advantage of DEP classification is that the particle migration velocity scales proportionally to $d_{{\text {P}}}^{2}$, whereas for DMA processes the electrophoretic migration velocity shows a significantly lower dependency. This allows potentially higher resolutions for DEP classification. The DMA migration velocity depends on the particle charging modus which can be unipolar diffusion or field charging or bipolar diffusion charging (typical for DMA). For bipolar diffusion charging the migration velocity of nanoparticles scales proportionally to $d_{{\text {P}}}^{-1}$ ($d_{{\text {P}}}<{100}\,{\hbox {nm}}$) while the scaling for bigger particles decreases. Field charging (corona charging) becomes significant for particles above 100 nm and the velocity scales proportionally to $d_{{\text {P}}}$ ($d_{{\text {P}}}>{1}\,{\upmu \hbox {m}}$) ^38^.
2. On the other hand, the DEP migration velocity is much lower than the electrophoretic migration velocity (approximately by four orders of magnitude at $d_{{\text {P}}} = {1}\,\upmu \hbox {m}$), and diffusion has a much higher impact on the resolution.

Hence, DEP classification is, compared to DMA, stronger limited by diffusion and less limited by the gas flow conditions.

## Conclusion

The possibilities and limitations of the classification of aerosol particles by dielectrophoresis (DEP) have been evaluated for concentric cylindrical electrodes with an annular gap in between serving as separation chamber. The observed dependencies may however be transferred to other geometries and thus have a general character. In fact, a systematic investigation of the dependencies of target particle diameter, resolution, and yield of the DEP classification on residence time, applied electric field strength, and pressure of the carrier gas was performed. In addition, the influence of particle diffusion on the DEP classification was also considered for the first time.

It was found that the target particle diameter $d_{{\text {P}}}^*$ scales with the residence time $t_{{\text {end}}}$ and the field strength *E* as $d_{{\text {P}}}^*\propto t_{{\text {end}}}^{-0.5}E^{-1}$, with the residence time being proportional to the ratio of classifier length to average gas flow velocity $t_{{\text {end}}} \propto L/u_{{\text {gas}}}$.

As expected and analogous to DMA applications, the resolution of DEP classification is limited either by diffusion or by the classification parameters. If diffusion is the limiting factor, which is the case for small particles and low flow ratios $\beta$, resolution is proportional to ${d_{{\text {P}}}^*}^{1.3}$ and can be increased by increasing the electric field strength. For bigger particles and larger flow ratios $\beta$ resolution is limited to a value that depends mainly on the flow ratio and the distance between the electrodes. For the case that the yield is 1, this limitation is given by $R_{{\text {cl}}}$. We defined as a characteristic value for this limitation the extended outlet slit length $s_{{\text {e}}}$ (which is an auxiliary value), so that the classifier limitation reads $R_{{\text {cl}}} = {\frac{(L+s_{{\text {e}}}/2)^{-0.5}}{L^{-0.5}-(L+s_{{\text {e}}})^{-0.5}}}$. The classification yield increases with the ratio of the extended outlet slit width $s_{{\text {e}}}$ to the diffusion induced broadening $\sigma _z$. As expected, resolution and yield exhibit opposite dependencies on the ratio $s_{{\text {e}}}/\sigma _{z}$.

Our simulations show the theoretical possibility to classify particles with diameters from about 20 nm to ${90}\,\upmu \hbox {m}$ when using pressures from 1 to 60 bar. Here, flow velocities between $u_\text {gas} = {0.1}\,\hbox {cm s}^{-1}$ (flow rate $Q={16}\,\hbox {mL s}^{-1}$) and $u_\text {gas} = {514}\,\hbox {cm s}^{-1}$ ($Q={800}\,\hbox {mL s}^{-1}$) were used. The throughput could be increased by changing the classifier geometry e.g. the classifier length. At this point, it is unclear to which extend these results can be achieved in praxis because the presented simulations did not account for particle interactions. Particle interactions increasingly occur at higher particle concentration and residence time. Nevertheless, at increased working pressures DEP classification can in principle cover a highly interesting particle size range from ${100}\,\hbox {nm}$ to ${10}\,\upmu \hbox {m}$, while being directly particle size-selective and particle charge independent.

## Supplementary information

Supplementary information.

## Data Availability

The datasets generated and analysed during the current study as well as the calculation algorithm are available from the corresponding author upon request.
