# Supplementary material for: Aerosol classification by dielectrophoresis: a theoretical study on spherical particles
Source: Sci Rep. 2020 Jun 30;10:10617. doi: 10.1038/s41598-020-67628-9 (PMC7327003; doi:10.1038/s41598-020-67628-9)
Supplement: Supplementary file 1 — Supplementary information. [file 41598_2020_67628_MOESM1_ESM.pdf]

Aerosol classification by dielectrophoresis: A theoretical study on spherical particles

Supplementary Information

Malte Lorenz Alfred P. Weber Michael Baune Jorg Thöming
Georg R. Pesch

1 Validation of the extended classifier model by comparison with DMA calculations

For validation of our new ”extended classifier model” we recalculated the transfer function of Hagwood et al. (1999) for classical DMA. Therefore we used the framework of the DEP classifier calculations (Sec. ”Model and Theory”) and applied an electrophoretic force $\mathbf{F}_{\mathrm{EP}}$ instead of the DEP force $\mathbf{F}_{\mathrm{DEP}}$ according to Hagwood et al. (1999):

$$\mathbf{F}_{\mathrm{EP}} = \frac{eC}{3\pi\mu d_{\mathrm{P}}}\mathbf{E}. \tag{1}$$

e is the charge of an electron, C the well known Cunningham slip correction factor, μ the dynamic viscosity of the ambient fluid, d_{P} the particle diameter and E the electric field. Our results (Fig. S1) show an excellent fit within a 3 % error margin of the peak voltage compared to the results of the original manuscript which were calculated by a Monte Carlo approach and by the analytical solution of Stolzenburg (1988). The difference in applied voltage can be explained by the fact that different correlations for the Cunningham slip correction factor and the gas flow field were used.

References

Hagwood, C., Sivathanu, Y., and Mulholland, G. (1999). The DMA transfer function with Brownian motion a trajectory/Monte-Carlo approach. *Aerosol Sci. Technol.*, 30(1):40–61.

Stolzenburg, M. R. (1988). *An Ultrafine Aerosol Size Distribution Measuring System.* Ph.d thesis, University of Minnesota.

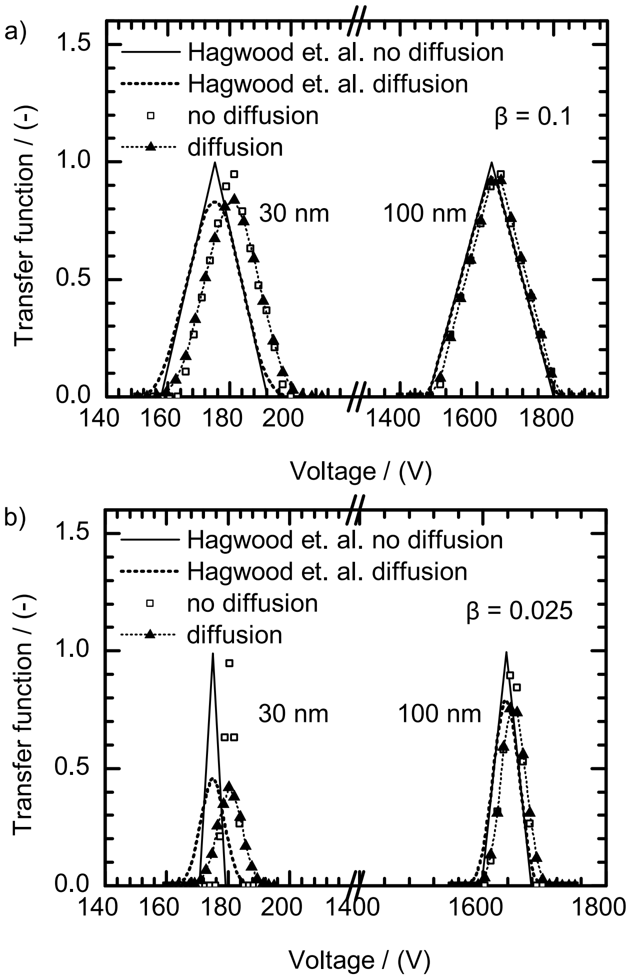

Figure S1: Comparison of our calculations to the work of Hagwood et al. (1999). Shown is the transfer function for particles of diameter $d_P = 30\,\mathrm{nm}$ and $d_P = 100\,\mathrm{nm}$ as a function of the applied voltage. Comparison was done for flow ratios of $\beta = 0.1$ (a) and $\beta = 0.025$ (b).
